# High-Sensitivity Cardiac Troponin I and Clinical Risk Scores in Patients With Suspected Acute Coronary Syndrome

**DOI:** 10.1161/CIRCULATIONAHA.118.036426

**Published:** 2018-10-15

**Authors:** Andrew R. Chapman, Kerrick Hesse, Jack Andrews, Kuan Ken Lee, Atul Anand, Anoop S. V. Shah, Dennis Sandeman, Amy V. Ferry, Jack Jameson, Simran Piya, Stacey Stewart, Lucy Marshall, Fiona E. Strachan, Alasdair Gray, David E. Newby, Nicholas L. Mills

**Affiliations:** 1 British Heart Foundation Centre for Cardiovascular Science, University of Edinburgh, United Kingdom (A.R.C., K.H., J.A., K.K.L., A.A., A.S.V.S., D.S., A.V.F., J.J., S.P., S.S., L.M., F.E.S., D.E.N., N.L.M.).; 2 Department of Emergency Medicine (A.G.), Royal Infirmary of Edinburgh, United Kingdom.; 3 Emergency Medicine Research Group of Edinburgh Research Group (A.G.), Royal Infirmary of Edinburgh, United Kingdom.; 4 Usher Institute of Population Health Sciences and Informatics, University of Edinburgh, UK (N.L.M.).

**Keywords:** high-sensitivity troponin, risk stratification

## Abstract

**Background:**

High-sensitivity cardiac troponin assays can help to identify patients who are at low risk of myocardial infarction in the emergency department. We aimed to determine whether the addition of clinical risk scores would improve the safety of early rule-out pathways for myocardial infarction.

**Methods:**

In 1935 patients with suspected acute coronary syndrome, we evaluated the safety and efficacy of 2 rule-out pathways alone or in conjunction with low-risk TIMI (Thrombolysis In Myocardial Infarction) (0 or 1), GRACE (Global Registry of Acute Coronary Events) (≤108), EDACS (Emergency Department Assessment of Chest Pain Score) (<16), or HEART (History, ECG, Age, Risk factors, Troponin) (≤3) scores. The European Society of Cardiology 3-hour pathway uses a single diagnostic threshold (99th percentile), whereas the High-STEACS (High-Sensitivity Troponin in the Evaluation of Patients With Acute Coronary Syndrome) pathway applies different thresholds to rule out (<5 ng/L) and rule in (>99th percentile) myocardial infarction.

**Results:**

Myocardial infarction or cardiac death during the index presentation or at 30 days occurred in 14.3% of patients (276/1935). The European Society of Cardiology pathway ruled out 70%, with 27 missed events giving a negative predictive value of 97.9% (95% CI, 97.1–98.6). The addition of a HEART score ≤3 reduced the proportion ruled out by the European Society of Cardiology pathway to 25% but improved the negative predictive value to 99.7% (95% CI, 99.0–100; *P*<0.001). The High-STEACS pathway ruled out 65%, with 3 missed events for a negative predictive value of 99.7% (95% CI, 99.4–99.9). No risk score improved the negative predictive value of the High-STEACS pathways, but all reduced the proportion ruled out (24% to 47%; *P*<0.001 for all).

**Conclusions:**

Clinical risk scores significantly improved the safety of the European Society of Cardiology 3-hour pathway, which relies on a single cardiac troponin threshold at the 99th percentile to rule in and rule out myocardial infarction. Where lower thresholds are used to rule out myocardial infarction, as applied in the High-STEACS pathway, risk scores halve the proportion of patients ruled out without improving safety.

**Clinical Trial Registration:**

URL: https://www.clinicaltrials.gov. Unique identifier: NCT01852123.

## Abstract 2

Supplemental Digital Content is available in the text.

**Clinical Perspective**

**What Is New?**

- We evaluate established rule-out pathways using high-sensitivity cardiac troponin testing to risk-stratify patients with suspected acute coronary syndrome and determine whether the addition of clinical risk scores (TIMI [Thrombolysis In Myocardial Infarction], GRACE [Global Registry of Acute Coronary Events], EDACS [Emergency Department Assessment of Chest Pain Score], and HEART [History, ECG, Age, Risk factors, Troponin]) is of benefit.
- The European Society of Cardiology 3-hour pathway missed >2 patients in every 100 tested, with safety markedly improved by inclusion of any risk score, all of which reduced the proportion of patients identified as low-risk.
- The High-STEACS (High-Sensitivity Troponin in the Evaluation of Patients With Acute Coronary Syndrome) pathway missed <1 in every 400 patients tested and did not benefit from the addition of clinical risk scores.

**What Are the Clinical Implications?**

- Clinicians should consider using a risk score if applying the European Society of Cardiology 3-hour pathway, which uses the 99th percentile to rule in and rule out myocardial infarction.
- Clinical risk scores do not improve the performance of pathways that apply low concentrations of cardiac troponin to risk-stratify patients, such as the High-STEACS pathway or the European Society of Cardiology 1-hour pathway.
- A prospective randomized controlled trial evaluating implementation of the High-STEACS pathway will provide further evidence of safety and efficacy in clinical practice and is due to report in early 2019.

Chest pain is a common presenting symptom in the emergency department, and although many patients require investigation for acute coronary syndrome, the majority have alternative diagnoses.^1–3^ Earlier identification of patients without myocardial infarction may improve patient experience and healthcare efficiency by reducing hospitalization for unnecessary investigation, but such strategies can only be implemented if safety is not compromised.

Several pathways permit the early rule-out of myocardial infarction. The European Society of Cardiology (ESC) 3-hour pathway uses the 99th percentile upper reference limit of a cardiac troponin assay to rule in and rule out myocardial infarction.^4^ However, recent observations have questioned whether this pathway provides adequate diagnostic performance in the era of high-sensitivity cardiac troponin testing.^5–7^ The precision of high-sensitivity assays at low concentrations has been exploited in the development of novel pathways that rule out myocardial infarction using thresholds <99th percentile. In an individual patient-level data meta-analysis of 22 457 patients, a cardiac troponin I threshold of <5 ng/L and a nonischemic ECG gave a negative predictive value (NPV) and sensitivity of 99.7% and 99.0% for myocardial infarction or cardiac death at 30 days, respectively.^8^ The same threshold has also been validated for the high-sensitivity cardiac troponin T assay in a recent pooled analysis.^9^ When this rule-out threshold was applied in a pathway that includes serial testing at 0 and 3 hours, 5-fold fewer patients were missed compared with a pathway that relies on the 99th percentile to rule out myocardial infarction.^6^

Clinical risk scores provide an alternative approach to identify patients at low risk of myocardial infarction who might be suitable for early discharge, but a number of uncertainties remain.^10^ New risk scores were developed^11^ or existing scores were incorporated into early rule-out pathways primarily to overcome the limitations of contemporary troponin assays.^12^ However, the role of clinical risk scores in pathways that incorporate high-sensitivity cardiac troponin testing is unclear, particularly in those pathways that apply different thresholds to rule out and rule in myocardial infarction. Here, we evaluate the safety and effectiveness of established early rule-out pathways with and without the addition of clinical risk scores.

## Methods

### Transparency and Openness Promotion

The analysis code for this study is available on request. Data can be made available to other researchers for the purposes of reproducing the results with a data sharing agreement.

### Study Population

Patients with suspected acute coronary syndrome were recruited from the emergency department of the Royal Infirmary of Edinburgh, a tertiary care hospital in Scotland, between June 1, 2013, and March 31, 2017, into a substudy of the High-STEACS trial(High-Sensitivity Troponin in the Evaluation of Patients With Acute Coronary Syndrome). All patients for whom the attending clinician requested cardiac troponin for suspected acute coronary syndrome were eligible for inclusion. We did not enroll patients with ST-segment elevation myocardial infarction, those who were unable to provide consent, or those from outside our region, to ensure complete follow-up. Blood samples were obtained at presentation and at 6 to 12 hours for high-sensitivity cardiac troponin testing as part of routine clinical care. Patients provided written informed consent for additional sampling at 3 hours, with the results of testing at this timepoint not used to guide patient care. This clinical trial was registered, approved by the national research ethics committee, and conducted in accordance with the Declaration of Helsinki.

### High-Sensitivity Cardiac Troponin I Assay

The Abbott ARCHITECT_*STAT*_ high-sensitive cardiac troponin I assay (Abbott Laboratories) is a 2-step chemoluminescent assay with a limit of detection of 1.2 ng/L and coefficient of variation of <10% at 6 ng/L.^13^ This assay performance has been independently validated across multiple centers under routine laboratory working conditions, with a reported interlaboratory coefficient of variation of 12.6% at 3.5 ng/L across 33 instruments.^14^ The upper reference limit 99th percentiles were determined in 4590 samples from healthy individuals as 16 ng/L for women and 34 ng/L in men,^15^ and from December 10, 2013, onward, these thresholds were used in clinical practice.

### Baseline Characteristics

Patient baseline characteristics, including chest pain characteristics, onset of symptoms, prior medical history, cardiovascular risk factors, medication, and clinical observations, in addition to investigations including serial 12-lead electrocardiography and cardiac imaging, were obtained from a dedicated case record form, patient questionnaire, and the electronic patient record (TrakCare, InterSystems). Hyperlipidemia or hypertension was defined as a history of the condition or by the use of lipid-lowering or antihypertensive therapies, respectively. Ischemic heart disease was defined as a history of angina, prior myocardial infarction, or prior coronary revascularization.

### Diagnostic Adjudication

The final diagnosis was adjudicated for all patients by 2 independent cardiologists, with consensus from a third cardiologist where there was discrepancy after review of all clinical information, both noninvasive and invasive investigations and outcomes from presentation to 30 days. Patients were classified as having type 1 myocardial infarction, type 2 myocardial infarction, or myocardial injury in accordance with the third universal definition of myocardial infarction as reported previously.^4,14^ Any high-sensitivity cardiac troponin I concentration above the sex-specific 99th percentile upper reference limit was considered evidence of myocardial necrosis. Type 1 myocardial infarction was defined as myocardial necrosis in the context of a presentation with symptoms suggestive of acute coronary syndrome or evidence of myocardial ischemia. Patients with symptoms or signs of myocardial ischemia because of increased oxygen demand or decreased supply (eg, tachyarrhythmia, hypotension, or anemia) secondary to an alternative pathology and myocardial necrosis were classified as type 2 myocardial infarction. Myocardial injury was defined as evidence of myocardial necrosis in the absence of any clinical features of myocardial ischemia. Further details of the adjudication process are available in the appendix in the online-only Data Supplement. Agreement for a diagnosis of type 1 myocardial infarction was good (κ=0.77; 95% CI, 0.69–0.84).

### Clinical Outcomes

The primary outcome was a composite of type 1 myocardial infarction or cardiac death during index presentation or at 30 days. We used regional and national registries in addition to individual patient follow-up at 30 days to ensure that follow-up was complete for the entire study population. All subsequent events were adjudicated using the same approach as for the index presentation. TrakCare software application (InterSystems Corporation) is a regional electronic patient record system that provides data on all hospital admissions to both tertiary and secondary care hospitals in the southeast of Scotland. All in-hospital and community deaths are recorded in a comprehensive national database, the General Register of Scotland. Cardiac death was defined as any death because of myocardial infarction, arrhythmia, or heart failure (*International Classification of Diseases, Tenth Revision*, codes I20-25, I34-37, I42, I43, I46, and I48-51).

### Clinical Pathways

**Figure 1.**
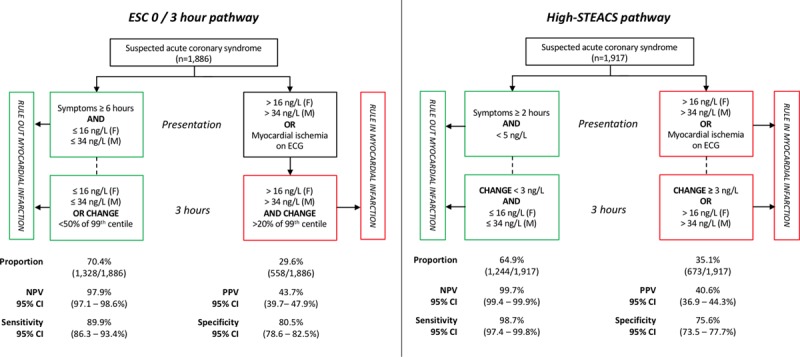
**Illustration of the ESC 3-hour and High-STEACS pathways.** ESC indicates European Society of Cardiology; F, female; High-STEACS, High-Sensitivity Troponin in the Evaluation of Patients With Acute Coronary Syndrome; M, male; NPV, negative predictive value; and PPV, positive predictive value.

We evaluated the safety and efficacy of the ESC 3-hour pathway and the High-STEACS pathway (Figure 1), with and without the addition of clinical risk scores, to rule out the composite outcome of index type 1 myocardial infarction and type 1 myocardial infarction or cardiac death at 30 days. These pathways were selected because they represent examples of approaches using troponin as a continuous variable or as a binary decision tool applying the 99th percentile alone. To improve generalizability, where samples were available, we also evaluated the ESC 1-hour pathway.

### ESC 3-Hour Pathway

The ESC 3-hour pathway rules out myocardial infarction in patients without ischemia on the ECG where cardiac troponin concentrations are <99th percentile at presentation in patients with symptoms for >6 hours. In patients with symptoms for <6 hours, a second troponin measurement is performed 3 hours from presentation, with myocardial infarction ruled out if cardiac troponin remains <99th percentile or is >99th percentile without a significant change in concentration.^4^ Previously published guidance from the ESC Working Group on Acute Cardiac Care recommends use of a change in cardiac troponin concentration >50% of the 99th percentile upper reference limit at 3 hours, where the initial concentration is ≤99th percentile or >20% when the initial concentration was >99th percentile.^16^ The ESC pathway recommends a GRACE (Global Registry of Acute Coronary Events) score of <140 in those who are pain free as a final step before discharge.

### High-STEACS Pathway

The derivation and validation of the High-STEACS pathway has been reported previously.^6^ This pathway was based on previous observations^8,14^ and utilizes a risk stratification threshold of 5 ng/L at presentation. Patients without myocardial ischemia on the ECG and cardiac troponin concentrations <5 ng/L at presentation are considered low risk, with myocardial infarction ruled out without further testing, unless they present early with symptom onset <2 hours from presentation where cardiac troponin is retested 3 hours after presentation. Patients with cardiac troponin concentrations ≥5 ng/L at presentation are retested at 3 hours. Myocardial infarction is ruled out at 3 hours if cardiac troponin concentrations are unchanged (Δ <3 ng/L) and remain ≤99th percentile.

### ESC 1-Hour Pathway

The ESC 1-hour pathway rules out myocardial infarction in patients without ischemia on the ECG where cardiac troponin concentrations are <2 ng/L at presentation and symptoms are present for >3 hours. In all other patients, myocardial infarction is ruled out if cardiac troponin concentrations are <5 ng/L at presentation with a change of <2 ng/L after 1 hour.^16^

### Clinical Risk Scores

**Figure 2.**
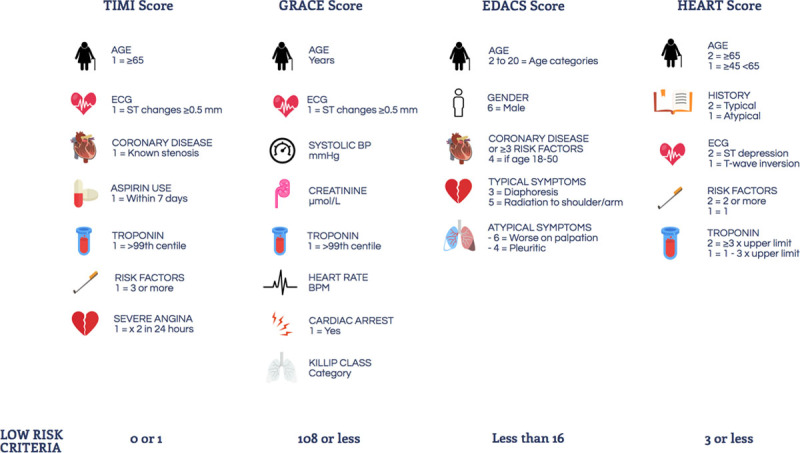
**Components of the TIMI, GRACE, EDACS, and HEART clinical risk scores.** The GRACE score algorithm is available online.^17^ BP indicates blood pressure; BPM, beats per minute; EDACS, Emergency Department Assessment of Chest Pain Score; GRACE, Global Registry of Acute Coronary Events; HEART, History, ECG, Age, Risk factors, Troponin; and TIMI, Thrombolysis In Myocardial Infarction.

We derived GRACE, TIMI (Thrombolysis In Myocardial Infarction), HEART (History, ECG, Age, Risk factors, Troponin), and EDACS (Emergency Department Assessment of Chest Pain Score) scores using prospectively collected clinical information documented in the case record form by the research nurse at the time of recruitment (Figure 2). We calculated the GRACE score for in-hospital death; this algorithm is available online.^17^ In line with prior recommendations, a GRACE score of ≤108 (estimated in-hospital mortality of <1%),^18^ a HEART score of ≤3,^11^ a TIMI score of 0 or 1,^19^ or an EDACS score of <16 was considered low-risk.^20^ For comparison, we provide the diagnostic performance of the HEART, GRACE, TIMI, and EDACS scores alone in the online-only Data Supplement.

### Sensitivity Analyses

We evaluated the NPV of all approaches for a primary outcome encompassing type 1 or type 2 myocardial infarction, myocardial injury, or cardiac death at 30 days. Because the High-STEACS pathway was derived in the first 1218 participants included in our dataset, we repeated our analyses and excluded these subjects. In a further analysis, we tested both pathways, excluding any patients who underwent invasive or noninvasive cardiac testing in ≤30 days of index presentation.

### Statistical Analysis

Baseline characteristics are summarized as mean (SD) or median (interquartile range) as appropriate. Where there were missing data for continuous variables, we imputed the median value. The primary outcome was the NPV of each pathway using the composite end point of index type 1 myocardial infarction, subsequent type 1 myocardial infarction, or cardiac death at 30 days.^14^ Because we estimated the NPV would approach 100%, we used a Bayesian approach with a Jeffreys prior (β distribution with both shape parameters equal to 0.5); this is more robust when CIs approach 0 or 1.^21^ We determined absolute (hs-TnI_*3hr*_–hs-TnI_*0hr*_) and relative ([(hs-TnI_*3hr*_–hs-TnI_*0hr*_) /hs-TnI_*0hr*_]x100) change in cardiac troponin concentration from presentation to 3 hours and determined sensitivity, specificity, and positive predictive value with 95% CIs using a Bayesian approach as per the NPV. We derived a weighted generalized score statistic to compare the NPV of each pathway with and without the addition of clinical risk scores.^22^ We evaluated pathway efficacy by determining the number of patients ruled out with the pathway alone or with the combination of pathway and risk score as a proportion of the total study population, with comparison by McNemar’s test for paired proportions. A 2-sided *P*<0.05 was considered statistically significant. All analyses were performed using R (Version 3.2.2).

## Results

**Figure 3.**
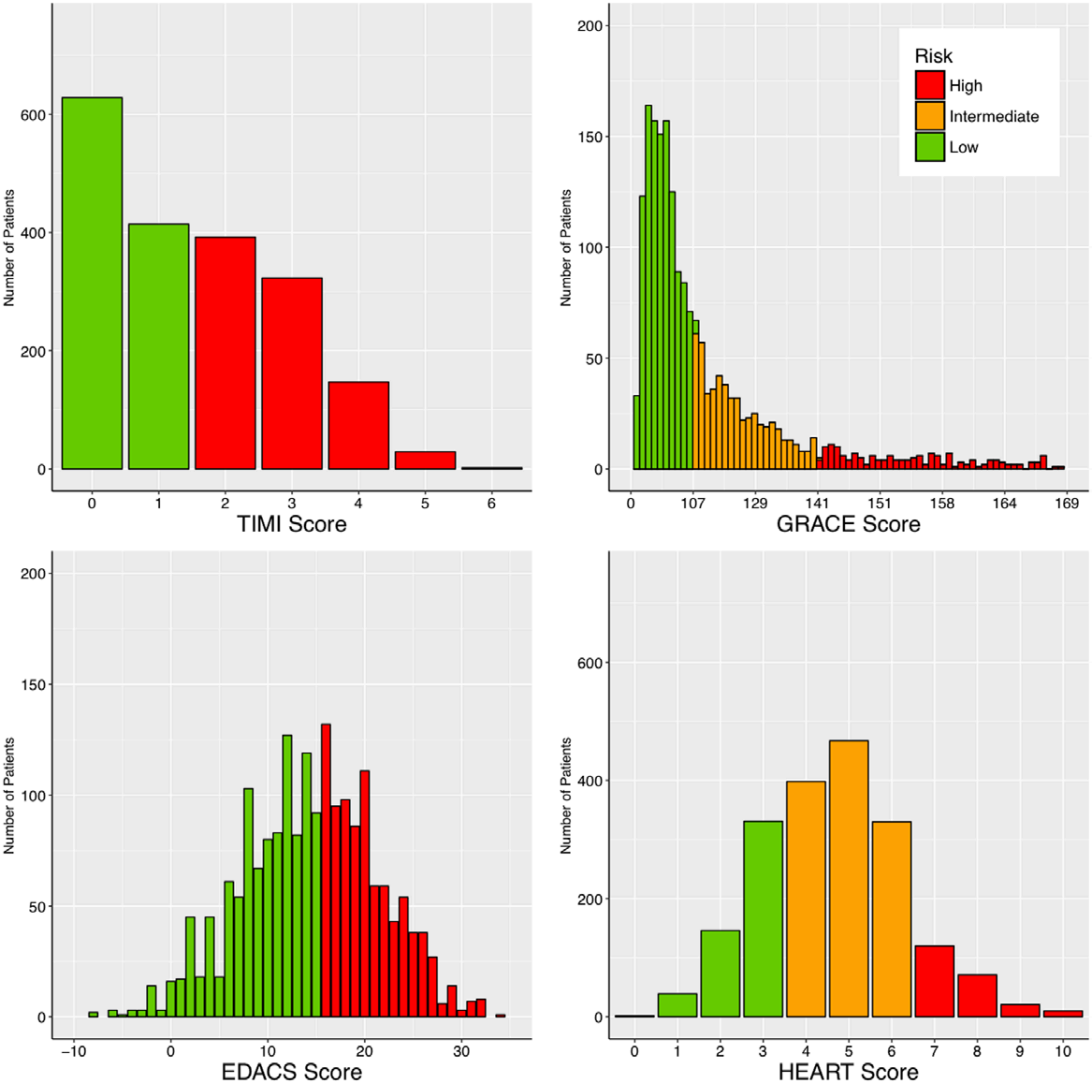
**Distribution of patients per risk category assigned by the TIMI, GRACE, EDACS, and HEART risk scores in the analysis population.** EDACS indicates Emergency Department Assessment of Chest Pain Score; GRACE, Global Registry of Acute Coronary Events; HEART, History, ECG, Age, Risk factors, Troponin; and TIMI, Thrombolysis In Myocardial Infarction.

We enrolled 1951 patients with suspected acute coronary syndrome, of whom 1935 had a cardiac troponin I result available from presentation (Tables 1 and 2 and Figure I in the online-only Data Supplement). The adjudicated diagnosis was type 1 myocardial infarction in 273 patients (14.1%), type 2 myocardial infarction in 77 patients (4%), and myocardial injury in 31 patients (1.6%), with 6 deaths from a cardiac cause at 30 days (Figure 3).

**Table 1.**
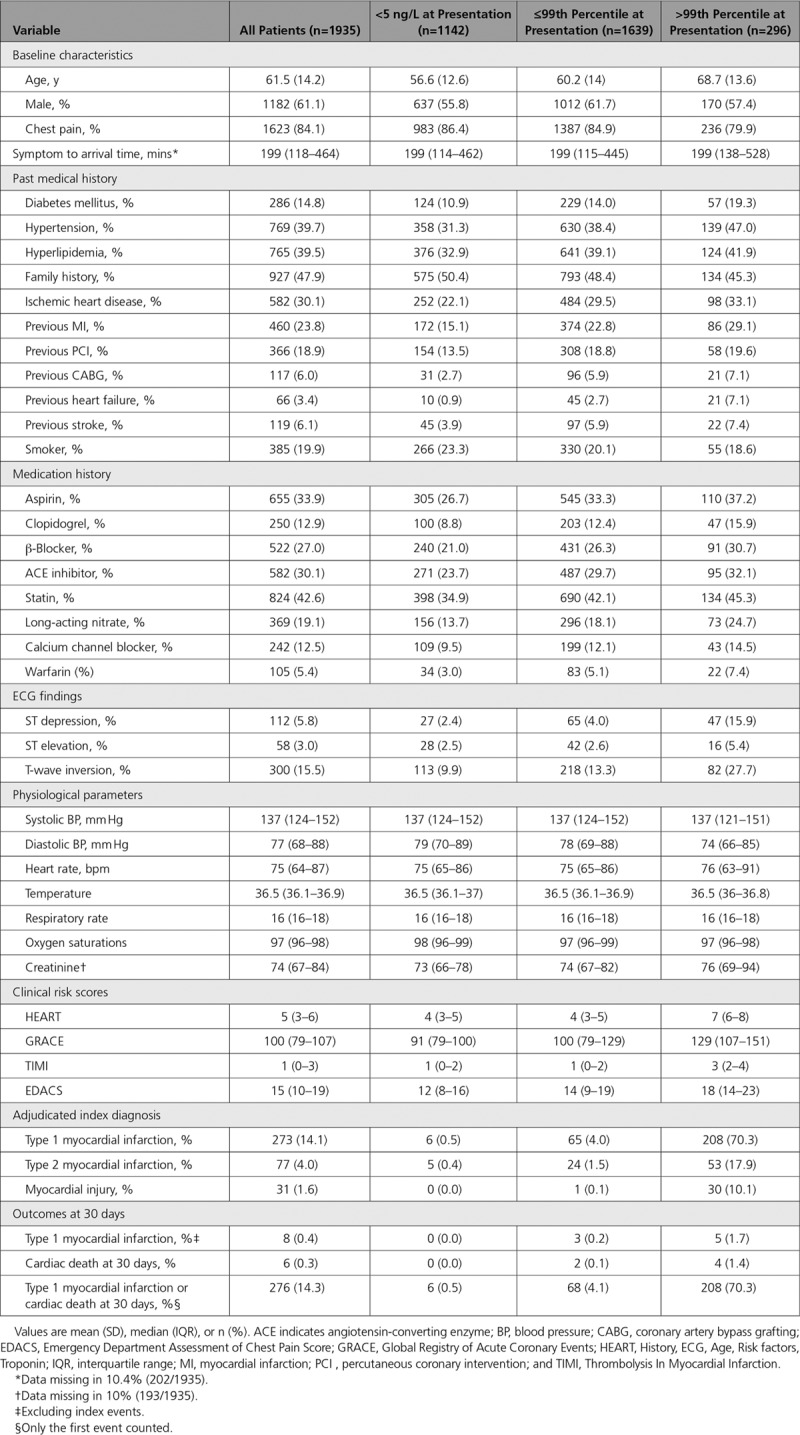
Baseline Characteristics of the Study Population Stratified by Troponin Concentration

**Table 2.**
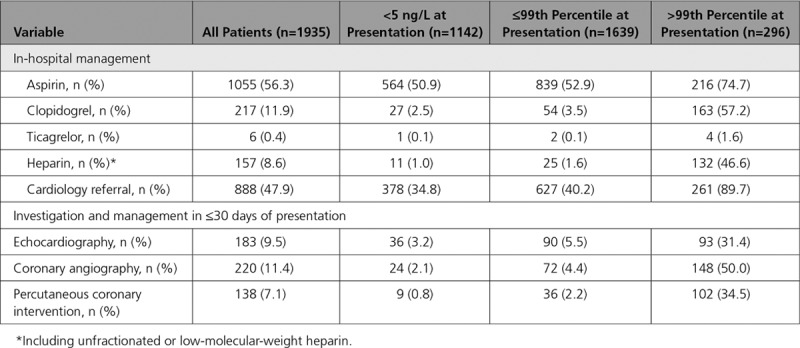
Investigation and Management of Patients Stratified by Troponin Concentration at Presentation

### ESC 3-Hour Pathway

A total of 1886 patients (97.5%) were included, with 49 patients excluded because of missing 3-hour samples, which were required for the ESC pathway (Figure 1). The ESC pathway identified 70% (1328/1886) of patients as low-risk, with 27 missed events (25 index type 1 myocardial infarction, 1 type 1 myocardial infarction, and 1 cardiac death at 30 days) (Table I in the online-only Data Supplement) for a NPV of 97.9% (95% CI, 97.1–98.6) and sensitivity of 89.9% (95% CI, 86.3–93.4) (Table 3).

**Table 3.**
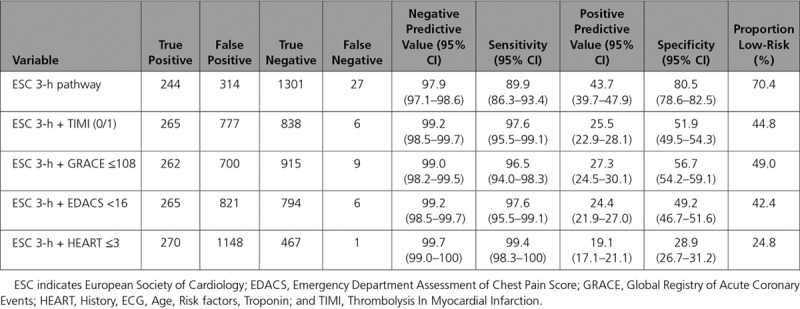
Diagnostic Metrics for the European Society of Cardiology 3-Hour Pathway With and Without Clinical Risk Scores

### ESC 3-Hour Pathway Plus Risk Scores

When a HEART score of ≤3 was applied alongside the ESC pathway, the proportion identified as low-risk fell from 70% to 24.8% (468/1886, *P*<0.001). However, the NPV improved to 99.7% (95% CI, 99.0–100; *P*<0.001). A similar improvement in safety was observed when an EDACS score of <16 was applied, with an NPV of 99.2% (95% CI, 98.5–99.7; *P*<0.001), identifying 42.4% as low-risk (800/1886, *P*<0.001). A TIMI score of 0 or 1 gave an NPV of 99.2% (95% CI, 98.5–99.7; *P*<0.001), and a GRACE score of ≤108 gave an NPV of 99.0% (95% CI, 98.2–99.5; *P*<0.001), with a reduction in the proportion identified as low-risk to 43.5% (844/1886, *P*<0.001) and 49% (924/1886, *P*<0.001) of patients, respectively. When a higher GRACE score of <140 was applied as recommended in the guideline, the NPV and sensitivity were lower at 98.1% (95% CI, 97.3–98.8) and 91.3% (95% CI, 87.8–94.4), respectively, with 23 missed index or 30-day events.

### High-STEACS Pathway

A total of 1917 patients (99.1%) were included, with 18 patients excluded because of missing 3-hour samples, which were required for the High-STEACS pathway (Figure 1). The High-STEACS pathway identified 64.9% (1244/1917) of patients as low-risk, with 3 missed events (2 index type 1 myocardial infarction and 1 type 1 myocardial infarction at 30 days) (Table II in the online-only Data Supplement) for an NPV of 99.7% (95% CI, 99.4–99.9) and sensitivity of 98.7% (95% CI, 97.4–99.8) (Table 4).

**Table 4.**
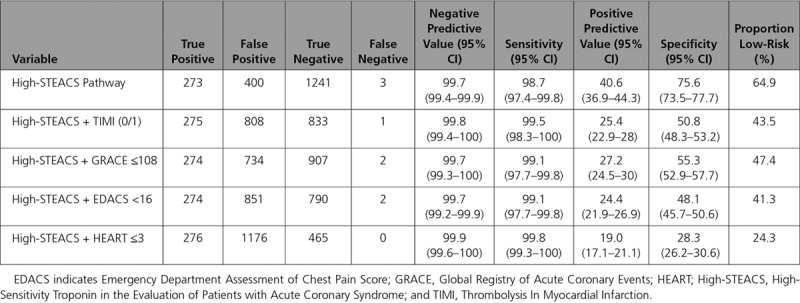
Diagnostic Metrics for the High-STEACS Pathway With and Without Clinical Risk Scores

### High-STEACS Pathway Plus Risk Scores

**Figure 4.**
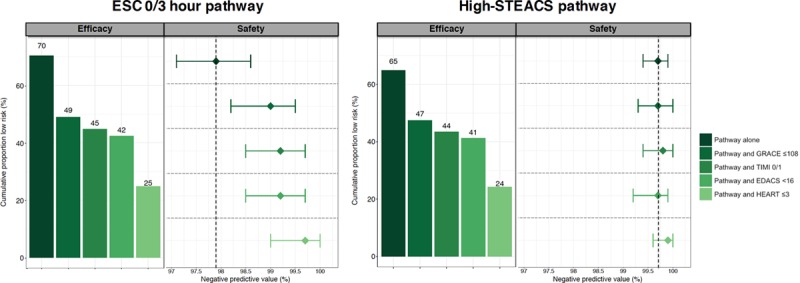
**Proportion of patients classified as low-risk on the basis of the ESC 3-hour or High-STEACS pathway alone or in combination with the TIMI, GRACE, EDACS, or HEART scores.** ESC indicates European Society of Cardiology; EDACS, Emergency Department Assessment of Chest Pain Score; GRACE, Global Registry of Acute Coronary Events; HEART, History, ECG, Age, Risk factors, Troponin; High-STEACS, High-Sensitivity Troponin in the Evaluation of Patients With Acute Coronary Syndrome; and TIMI, Thrombolysis In Myocardial Infarction.

When a HEART score ≤3 was applied alongside the High-STEACS pathway, the proportion identified as low-risk fell to 24.3% (465/1917, *P*<0.001). There was no improvement in the NPV (99.9%; 95% CI, 99.6–100; *P*=0.083). Similarly, no improvements in NPV were observed when the High-STEACS pathway was applied in conjunction with an EDACS score of <16 (NPV, 99.7%; 95% CI, 99.2–99.9; *P*=0.912), a TIMI score of 0 or 1 (NPV, 99.8%; 95% CI, 99.4–100; *P*=0.313), or a GRACE score of ≤108 (NPV, 99.7%; 95% CI, 99.3–100; *P*=0.815). All risk scores reduced the proportion of patients identified as low-risk (EDACS 41%, TIMI 44%, and GRACE 47%; *P*<0.001 for all) (Figure 4).

### Sensitivity Analyses

We repeated our analyses of the High-STEACS and ESC 3-hour pathways for a composite end point, including type 1 or type 2 myocardial infarction or myocardial injury with similar performance observed (Tables III and IV in the online-only Data Supplement). Because the High-STEACS pathway was derived in the first 1218 participants of this cohort study, we repeated our analyses and excluded these patients, with no differences observed in diagnostic performance (Table V in the online-only Data Supplement). In a further sensitivity analysis, we excluded any patients who underwent invasive or noninvasive cardiac testing and observed no differences in safety or efficacy (Table VI in the online-only Data Supplement). The diagnostic metrics for each risk score alone are provided in Table VII in the online-only Data Supplement.

### ESC 1-Hour Rule-Out Pathway

Where samples were available at presentation and 1 hour (406/1935), we evaluated the performance of the ESC 1-hour rule-out pathway (Table VIII in the online-only Data Supplement). In this population, the prevalence of the primary outcome was 8.1% (33/406). The ESC 1-hour pathway identified 37.7% (153/406) of patients as low-risk at presentation and 71.4% (290/406) of patients as low-risk at 1 hour, with no missed cases, for an NPV of 99.8% (95% CI, 99.3–100) and sensitivity of 98.5% (95% CI, 94.4–100). Because no cases were missed, no risk score improved safety, but all significantly reduced the proportion identified as low-risk. When we applied the High-STEACS pathway in the subgroup of patients with 1-hour samples available, there were also no missed events.

## Discussion

In a prospective observational cohort study of patients with suspected acute coronary syndrome, we evaluated the performance of high-sensitivity cardiac troponin testing in the ESC 3-hour pathway and the High-STEACS pathway, which applies a lower threshold to rule out myocardial infarction with and without the addition of clinical risk scores. We make a number of clinically relevant observations.

When used in isolation, the ESC pathway identifies a high proportion of patients as low-risk, but the NPV and sensitivity were poor, missing 27 index or 30-day events. The addition of a clinical risk score markedly improves safety, with the combination of ESC pathway and a HEART score ≤3 missing just 1 index event. However, this strategy identifies 3-fold fewer patients as low-risk. Conversely, the High-STEACS pathway incorporates a lower threshold of 5 ng/L to rule out myocardial infarction and has both a high NPV and sensitivity, missing just 3 index or 30-day events and identifying two-thirds of patients as low-risk. There was no improvement in diagnostic performance when the High-STEACS pathway was applied in conjunction with the TIMI, GRACE, EDACS, or HEART scores, but there was a 2- to 3-fold reduction in the proportion of patients identified as low-risk.

The ESC 3-hour pathway was first introduced in the 2011 NSTE-ACS guideline (Non-ST Elevation Acute Coronary Syndrome) and has been a central component of our evaluation of patients with suspected acute coronary syndrome.^4,18^ This pathway was devised in an era of contemporary cardiac troponin assays, where the upper reference limit was ≤5-fold higher than that applied with current generation high-sensitivity assays.^23^ It is perhaps unsurprising that when evaluated with more sensitive assays, the diagnostic performance of this pathway is worse. Several recent studies have demonstrated a low NPV, with diagnostic sensitivities <95%, well below the level deemed clinically acceptable.^5–7^ Although the ESC guideline includes a low-risk GRACE score (≤140) as a final step before discharge, there is a lack of clarity as to the intended strategy for this approach in clinical practice. In the present analysis, we demonstrate that a GRACE score ≤140 is not effective, but a more conservative GRACE score of ≤108 does improve the NPV and sensitivity, although the latter remained at 96%.

The GRACE and TIMI scores were derived in patients with confirmed myocardial infarction and were designed to guide prognostication and management. These scores have been extrapolated for use as risk stratification tools in patients with suspected acute coronary syndrome, and both improved the performance of the ESC pathway. In contrast, both the HEART and EDACS scores were derived and validated in patients with suspected, not confirmed, myocardial infarction.

The HEART score has been shown to perform better than GRACE and TIMI in patients with suspected acute coronary syndrome.^11^ In this analysis, we demonstrate the greatest improvement in the safety of the ESC pathway when a HEART score of ≤3 was included. The ESC pathway and the HEART score appear synergistic, with the combination of strategies offering a better safety profile than either in isolation. Our observation is consistent with a recent meta-analysis of 11 217 patients with suspected acute coronary syndrome, in whom a HEART score ≤3 gave a sensitivity of 96.7%.^24^ The current HEART score uses troponin as a categorical variable based on multiples of the upper reference limit. One option to improve the performance of the HEART score further would be to incorporate high-sensitivity cardiac troponin concentrations as a continuous marker of risk^4^ and to harness rather than omit this information to aid risk stratification. Similar improvements in the safety of the ESC pathway were observed when applied in conjunction with a low-risk EDACS score. However, this approach identified almost twice as many patients as low-risk. This observation may influence clinicians when considering which approach to implement in practice.

The High-STEACS pathway applies a cardiac troponin threshold of <5 ng/L in conjunction with a nonischemic ECG as an initial risk stratification step, with serial testing at 3 hours in all other patients. This pathway performs well in patients with suspected acute coronary syndrome, and we demonstrate no improvement in safety with the addition of clinical risk scores. When applied in isolation, the High-STEACS pathway ruled out 1244 patients with 3 missed events (a miss rate of <1 in 400 patients). The safety of pathways incorporating low concentrations of cardiac troponin for risk stratification is high and not improved by additional risk scores. One of the reasons this approach is so effective is that cardiac troponin concentrations are increased in patients with risk factors for acute coronary syndrome (such as hyperlipidemia, hypertension, or renal impairment) or in those with subclinical coronary or structural heart disease that may not be evident at presentation to the emergency department.^13,25–27^

There are several alternatives to the 2 rule-out pathways presented in this analysis.^28^ The ESC introduced 0- and 1-hour rule-out pathways in their 2015 guideline.^4^ This pathway has excellent diagnostic performance and has been validated in a number of settings, including a subgroup analysis of the present study.^29–33^ However, the practicality of delivering presentation troponin results and obtaining serial testing at 1 hour may be challenging in many healthcare settings. Until point-of-care solutions facilitate rapid turnaround times with similar test characteristics to laboratory troponin assays, safe alternative pathways with serial testing at 2 or 3 hours are required. In such settings, we believe the ESC 3-hour pathway should be applied with a clinical risk score. Alternatively, the EDACS score alone provides excellent safety and efficacy when applied with a nonischemic ECG and serial cardiac troponin testing at 0 and 2 hours, as recommended by the authors (Table VII in the online-only Data Supplement).^20^

In settings where high-sensitivity cardiac troponin I testing is available, the High-STEACS pathway is a more effective alternative approach, identifying more patients as low-risk without compromising safety. The performance of this pathway is currently being evaluated in a stepped-wedge cluster randomized trial of ≈30 000 patients in Scotland (NCT03005158).

There are important limitations to the data presented. This is a single-center observational cohort study. However, as a large tertiary cardiology center, we believe our findings are likely to be generalizable. The High-STEACS pathway was derived in the first 1218 patients included in this population. Although the performance was identical in our sensitivity analysis excluding these patients, further external validation studies are required. Because the majority of patients underwent serial sampling at 3 hours (Table IX in the online-only Data Supplement), we were only able to evaluate the ESC 1-hour pathway in a minority of patients. Because this strategy includes low concentrations of cardiac troponin to risk-stratify patients, it is likely that the performance would be similar to the High-STEACS pathway in the full population. To date, no comparison studies of these approaches have been undertaken. We were not able to undertake evaluation of other validated rule-out pathways such as ADAPT (Accelerated Diagnostic Protocol to Assess Patients With Chest Pain Symptoms Using Contemporary Troponins as the Only Biomarker) or T-MACS (Troponin only Manchester Acute Coronary Syndrome score). Our analysis of the ESC 3-hour pathway focused on high-sensitivity cardiac troponin I. However, similar findings have been documented in studies of high-sensitivity cardiac troponin T.^5^ Finally, as with all observational cohort studies, no patients were discharged on the basis of the pathways evaluated, and differences in management may have influenced outcomes. Although we did not have information on rates of exercise tolerance testing or nuclear testing, patients with low troponin concentrations were less likely to undergo transthoracic echocardiography or invasive coronary angiography (Table 2). When we repeated the primary analysis removing all patients who underwent these investigations (Table VI in the online-only Data Supplement), we observed no reduction in safety. Nevertheless, the results of implementation studies are necessary to guide clinical practice and future guidelines.

## Conclusions

Clinical risk scores significantly improve the safety of the ESC 3-hour pathway that relies on the 99th percentile to rule out myocardial infarction. Where lower cardiac troponin concentrations are used to rule out myocardial infarction, as applied in the High-STEACS pathway, risk scores halve the proportion of patients ruled out without further improvements in safety.

## Sources of Funding

This work was supported by the British Heart Foundation (SP/12/10/29922 and PG/15/51/31596). A.R.C., N.L.M., and D.E.N. are supported by a Clinical Research Training Fellowship (FS/16/75/32533), a Butler Senior Clinical Research Fellowship (FS/16/14/32023), and a Chair (CH/09/002) award from the British Heart Foundation. A.A. is supported by a research fellowship from Chest Heart and Stroke Scotland (15/A163). D.E.N. is the recipient of a Wellcome Trust Senior Investigator Award (WT103782AIA). The funders had no role in the design or conduct of the study; the collection, analysis, and interpretation of data; or the preparation, review, or approval of the manuscript.

## Disclosures

A.R.C. has received honoraria from Abbott Diagnostics and AstraZeneca. A.A. and A.S.V.S. have received honoraria from Abbott Diagnostics. N.L.M. has acted as a consultant for Abbott Diagnostics, Beckman-Coulter, Roche, and Singulex. The other authors report no conflicts of interest

## Supplementary Material
