# Supplementary material for: High-Sensitivity Cardiac Troponin I and Clinical Risk Scores in Patients With Suspected Acute Coronary Syndrome
Source: Circulation. 2018 Oct 15;138(16):1654–65. doi: 10.1161/CIRCULATIONAHA.118.036426 (PMC6200389; doi:10.1161/CIRCULATIONAHA.118.036426)
Supplement: Supplementary file 1 [file cir-138-1654-s001.pdf]

DATA SUPPLEMENT

High-Sensitivity Cardiac Troponin I and Clinical Risk Scores in Patients With Suspected Acute Coronary Syndrome

Chapman et al: High-Sensitivity Cardiac Troponin and Risk Scores

Andrew R. Chapman, MD;[1] Kerrick Hesse, MD;[1] Jack Andrews, MD;[1] Kuan Ken Lee, MD;[1] Atul Anand, MD;[1] Anoop S.V. Shah, MD;[1] Dennis Sandeman, MSc;[1] Amy V. Ferry, BSc;[1] Jack Jameson;[1] Simran Piya;[1] Stacey Stewart, MN;[1] Lucy Marshall, MSc;[1] Fiona E. Strachan, PhD;[1] Alasdair Gray, MD;[2,3] David E. Newby, MD, PhD;[1] Nicholas L. Mills, MD, PhD[1]

[1]BHF Centre for Cardiovascular Science, University of Edinburgh, United Kingdom
[2]Department of Emergency Medicine, Royal Infirmary of Edinburgh, United Kingdom
[3]EMERGE Research Group, Royal Infirmary of Edinburgh, United Kingdom

Correspondence to
Dr Andrew R. Chapman
BHF/University Centre for Cardiovascular Science
Chancellor's Building
University of Edinburgh
Edinburgh EH16 4SB
United Kingdom
Phone: +44 131 242 6431
Fax: +44 131 242 6379
E-mail: a.r.chapman@ed.ac.uk
Twitter: @chapdoc1

Supplemental Figure 1. Flow diagram illustrating identification of the study population

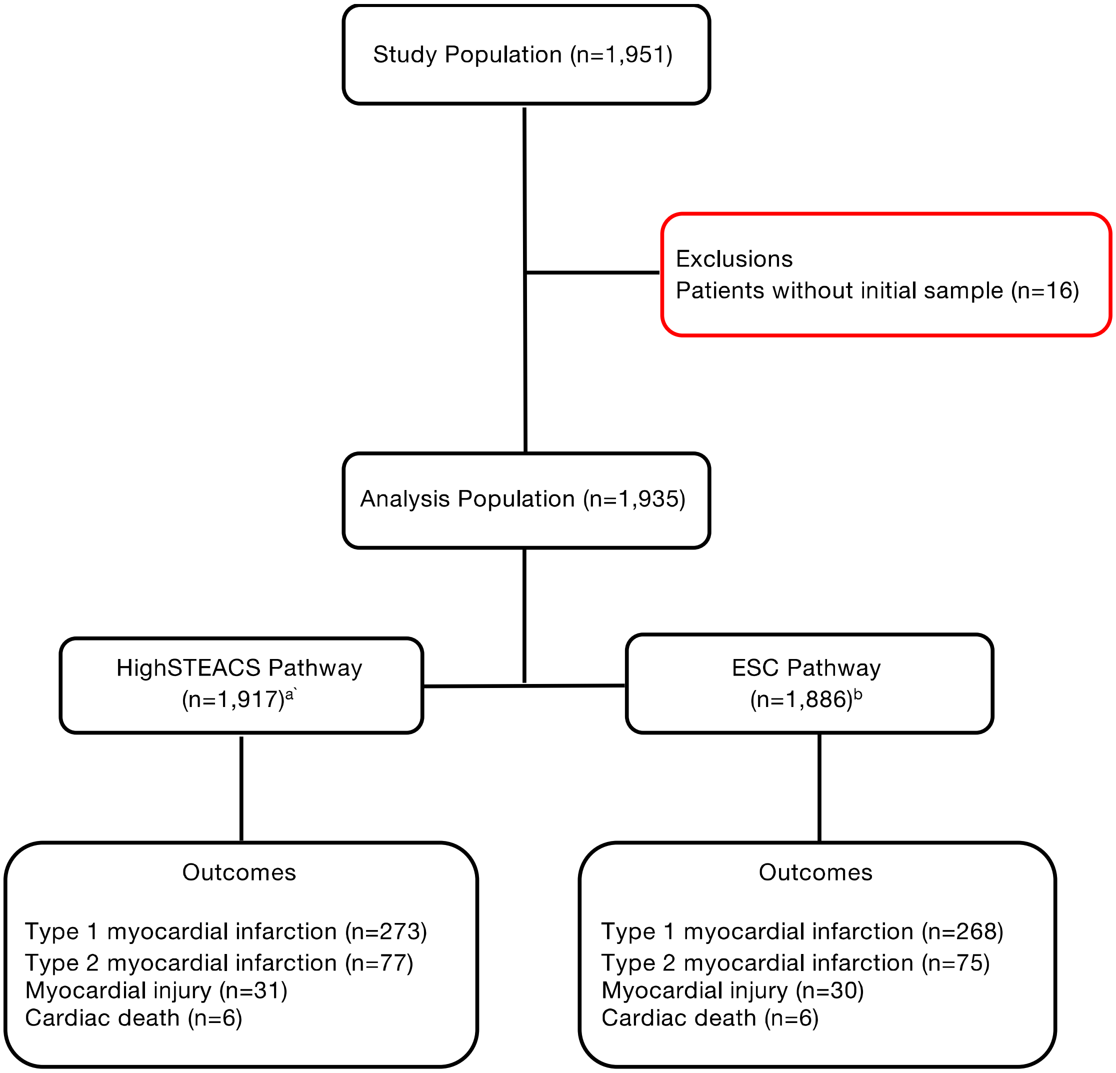

[a] 18 patients excluded as missing required serial sample for HighSTEACS pathway
[b] 49 patients excluded as missing required serial sample for ESC pathway

Supplemental Table 1. Summary of missed index or 30-day events using the ESC 3 hour pathway

Age	Gender	Time since symptom onset (Minutes)	Troponin concentration, ng/L<br><br>Presentation	Troponin concentration ng/L<br><br>3 hours	Troponin concentration ng/L<br><br>Peak	Presenting Symptom	Diagnosis	TIMI Score	GRACE Score	EDACS Score	HEART Score
82	F	86	11	15	26	Chest pain	Index Type 1 MI	2	169	17	5
62	M	70	27	32	43	Chest pain	Index Type 1 MI	3	107	24	5
73	F	150	35	34	37	Chest pain	Index Type 1 MI	2	129	14	6
64	M	199	42	45	48	Chest pain	Index Type 1 MI	4	129	16	6
89	M	317	68	74	934	Chest pain	Index Type 1 MI	4	214	27	7
85	M	150	16	12	-	Chest pain	Cardiac death (30d)	3	107	23	6
80	F	191	17	17	15	Chest pain	Index Type 1 MI	3	129	19	7
79	M	107	37	35	28	Chest pain	Index Type 1 MI	3	173	22	7
73	M	180	26	29	41	Chest pain	Index Type 1 MI	4	107	24	5
82	M	126	19	20	22	Chest pain	Type 1 MI (30d)	4	169	24	6
66	M	89	12	31	202	Chest pain	Index Type 1 MI	3	107	18	5
56	M	202	8	14	307	Chest pain	Index Type 1 MI	3	65	10	5
65	F	57	47	44	-	Chest pain	Index Type 1 MI	2	79	10	6
88	F	82	18	15	12	Chest pain	Index Type 1 MI	4	141	24	6
66	M	305	22	36	50	Palpitations	Index Type 1 MI	3	79	22	5
60	M	295	14	14	170	Chest pain	Index Type 1 MI	3	65	14	4
88	F	222	15	19	-	Chest pain	Index Type 1 MI	3	129	20	5
89	F	165	16	18	24	Chest pain	Index Type 1 MI	1	129	20	6
80	F	144	21	21	-	Jaw pain	Index Type 1 MI	2	129	16	6

58	M	112	20	33	35	Chest pain	Index Type 1 MI	0	95	14	4
54	M	96	22	32	36	Chest pain	Index Type 1 MI	1	85	16	3
58	F	135	20	21	19	Chest pain	Index Type 1 MI	2	107	16	6
72	M	199	56	58	-	Chest pain	Index Type 1 MI	2	129	25	5
57	M	458	33	80	144	Chest pain	Index Type 1 MI	0	65	14	4
70	M	375	17	160	2583	Chest pain	Index Type 1 MI	4	84	18	6
85	F	1616	7	12	19	Chest pain	Index Type 1 MI	1	129	18	5
61	M	790	15	82	635	Chest pain	Index Type 1 MI	0	65	16	4

Supplemental Table 2. Summary of missed index or 30-day events using the High-STEACS pathway

Age	Gender	Time since symptom onset (Minutes)	Troponin concentration, ng/L<br><br>Presentation	Troponin concentration ng/L<br><br>3 hours	Troponin concentration ng/L<br><br>Peak	Presenting Symptom	Diagnosis	TIMI Score	GRACE Score	EDACS Score	HEART Score
82	M	126	19	20	22	Chest pain	Type 1 MI (30d)	4	169	24	6
60	M	295	14	14	170	Chest pain	Index Type 1 MI	3	65	14	4
64	F	134	1	54	19066	Chest pain	Index Type 1 MI	0	79	15	4

Supplemental Table 3. Diagnostic metrics for the European Society of Cardiology 0h / 3h pathway with and without clinical risk scores for a composite outcome of type 1 or type 2 myocardial infarction or cardiac death at 30 days

	True Positive	False Positive	True Negative	False Negative	Negative predictive value (95%CI)	Sensitivity (95%CI)	Positive predictive value (95%CI)	Specificity (95% CI)	Proportion low risk (%)
**ESC Pathway**	327	231	1279	49	96.3 (95.2-97.2)	86.9 (83.4-90.2)	58.6 (54.5-62.6)	84.7 (82.9-86.5)	70.4
**ESC Pathway + TIMI (0/1)**	368	674	836	8	99.0 (98.2-99.6)	97.7 (96.0-99.0)	35.3 (32.5-38.3)	55.4 (52.8-57.9)	44.8
**ESC Pathway + GRACE ≤108**	362	600	910	14	98.4 (97.5-99.1)	96.2 (94.0-97.9)	37.6 (34.6-40.7)	60.3 (57.8-62.7)	49.0
**ESC Pathway + EDACS <16**	363	723	787	13	98.3 (97.3-99.1)	96.4 (94.3-98.0)	33.4 (30.7-36.3)	52.1 (49.6-54.6)	42.4
**ESC Pathway + HEART ≤3**	374	1044	466	2	99.5 (98.6-99.9)	99.3 (98.3-99.9)	26.4 (24.1-28.7)	30.9 (28.6-33.2)	24.8

ESC – European Society of Cardiology, TIMI – Thrombolysis In Myocardial Infarction, GRACE – Global Registry of Acute Coronary Events, CI – confidence interval

Supplemental Table 4. Diagnostic metrics for the High-STEACS pathway with and without clinical risk scores for a composite outcome of type 1 or type 2 myocardial infarction or myocardial injury or cardiac death at 30 days

	True Positive	False Positive	True Negative	False Negative	Negative predictive value (95%CI)	Sensitivity (95%CI)	Positive predictive value (95%CI)	Specificity (95% CI)	Proportion low risk (%)
**High-STEACS Pathway**	378	295	1238	6	99.5 (99.0-99.8)	98.3 (97.0-99.5)	56.2 (52.4-59.9)	80.7 (78.8-82.7)	64.9
**High-STEACS + TIMI (0/1)**	382	701	832	2	99.7 (99.2-100)	99.4 (98.3-99.9)	35.3 (32.5-38.2)	54.3 (51.8-56.8)	43.5
**High-STEACS + GRACE ≤108**	381	627	906	3	99.6 (99.1-99.9)	99.1 (97.9-99.8)	37.8 (34.8-40.8)	59.1 (56.6-61.5)	47.4
**High-STEACS + EDACS <16**	382	743	790	2	99.7 (99.2-99.9)	99.4 (98.3-99.9)	34.0 (31.3-36.8)	51.5 (49.0-54.0)	41.3
**High-STEACS + HEART ≤3**	384	1068	465	0	99.9 (99.6-100)	99.9 (99.5-100)	26.5 (24.2-28.8)	30.3 (28.1-32.7)	24.3

High-STEACS = High-Sensitivity Troponin in the Evaluation of patients with Acute Coronary Syndrome, TIMI – Thrombolysis In Myocardial Infarction, GRACE – Global Registry of Acute Coronary Events, CI – confidence interval

Supplemental Table 5. Diagnostic metrics for High-STEACS pathway excluding the first 1,218 participants in whom the pathway was derived

	True Positive	False Positive	True Negative	False Negative	Negative predictive value (95%CI)	Sensitivity (95%CI)	Positive predictive value (95%CI)	Specificity (95% CI)	Proportion low risk (%)
**High-STEACS Pathway**	80	97	463	1	99.7 (99.3-100)	98.2 (95.3-100)	45.2 (38.0-52.6)	82.6 (79.5-85.7)	72.4

Supplemental Table 6. Diagnostic metrics for High-STEACS and the ESC pathway excluding all patients who underwent cardiac testing

	True Positive	False Positive	True Negative	False Negative	Negative predictive value (95%CI)	Sensitivity (95%CI)	Positive predictive value (95%CI)	Specificity (95% CI)	Proportion low risk (%)
**High-STEACS Pathway**	74	326	1176	1	99.9 (99.6-100)	98.0 (94.9-100)	18.6 (14.9-22.5)	78.3 (76.2-80.3)	74.6
**ESC Pathway**	64	252	1225	11	99.1 (98.5-99.5)	84.9 (76.8-92.5)	20.3 (16.1-24.9)	82.9 (81.0-84.8)	79.6

Of 1,594 patients who did not undergo cardiac testing, serial samples required for the High-STEACS and the ESC Pathways were missing in 17 and 42 patients respectively.

Supplemental Table 7. Diagnostic metrics for HEART, GRACE, TIMI and EDACS scores alone

	True Positive	False Positive	True Negative	False Negative	Negative predictive value (95%CI)	Sensitivity (95%CI)	Positive predictive value (95%CI)	Specificity (95% CI)	Proportion low risk (%)
**TIMI 0/1**	213	674	967	63	93.8 (92.3-95.2)	77.1 (72.0-81.8)	24.0 (21.3-26.9)	58.9 (56.5-61.3)	53.7
**GRACE ≤108**	201	566	1075	75	93.4 (91.9-94.8)	72.7 (67.4-77.8)	26.2 (23.2-29.4)	65.5 (63.2-67.8)	60.0
**EDACS <16 ***	273	834	767	3	99.5 (99.0-99.9)	98.7 (97.1-99.7)	24.7 (22.2-27.3)	47.9 (45.5-50.4)	41.0
**HEART ≤3**	268	1136	505	8	98.3 (97.1-99.3)	96.9 (94.6-98.6)	19.1 (17.1-21.2)	30.8 (28.6-33.0)	26.8

** When EDACS is applied in isolation, the following low risk criteria are recommended: 1) EDACS Score <16, 2) No myocardial ischaemia on the ECG and 3) troponin concentrations are ≤99th centile at 0 and 2 hours. Than M et al. Emerg Med Australas. 2014;26:34-44.*

TIMI – Thrombolysis In Myocardial Infarction, GRACE – Global Registry of Acute Coronary Events, CI – confidence interval

Supplemental Table 8. Diagnostic metrics for the ESC 1-hour pathway with and without clinical risk scores

	**True Positive**	**False Positive**	**True Negative**	**False Negative**	**Negative predictive value (95% CI)**	**Sensitivity (95% CI)**	**Positive predictive value (95% CI)**	**Specificity (95% CI)**	**Proportion low risk (%)**
**ESC 1-hour pathway**	33	83	290	0	99.8 (99.3-100)	98.5 (94.4-100)	28.6 (20.8-37.1)	77.7 (73.4-81.8)	71.4
**ESC 1-hour + TIMI (0/1)**	33	149	224	0	99.8 (99.1-100)	98.5 (94.4-100)	18.3 (13.1-24.2)	60.0 (55.0-64.9)	55.2
**ESC 1-hour + GRACE ≤108**	33	134	239	0	99.8 (99.2-100)	98.5 (94.4-100)	19.9 (14.3-26.3)	64.0 (59.1-68.8)	58.9
**ESC 1-hour + EDACS <16**	33	151	222	0	99.8 (97.2-100)	98.5 (94.4-100)	18.1 (12.9-24.0)	59.5 (54.5-64.4)	54.7
**ESC 1-hour + HEART ≤3**	33	239	134	0	99.6 (98.6-100)	98.5 (94.4-100)	12.3 (8.7-16.4)	36.0 (31.2-40.9)	33.0

TIMI – Thrombolysis In Myocardial Infarction, GRACE – Global Registry of Acute Coronary Events, CI – confidence interval

Supplemental Table 9. Available blood samples and median time of sampling in the High-STEACS substudy

	**Presentation**	**3 hours from presentation**	**6 – 12 hours from presentation**	**1 hour from first sample**
**Time from arrival to sample (minutes)**<br><br>*Median (IQR)*	28 (15 – 46)	176 (146-206)	416 (216-605)	65 (60 – 73)
**Proportion of patients with samples available**<br><br>*% (n)*	100 (1,935)	94.9 (1,837)	51.2 (990)	21 (406)
**Sample rules**	All samples included	All samples included	All samples included	Included if ≥ 30 and ≤90 minutes from time of first sample

Appendix 1. Additional information on diagnostic adjudication

Criteria for adjudication of patients with myocardial necrosis

**Type 1 myocardial infarction**	Myocardial necrosis (any cardiac troponin I [cTnI] concentration above the upper reference limit) with rise and or fall in cTnI concentration where serial testing was available AND symptoms OR signs of myocardial ischaemia
**Type 2 myocardial infarction**	Myocardial necrosis (any cTnI concentration above the upper reference limit) with rise and or fall in cTnI concentration where serial testing was available AND symptoms OR signs of myocardial ischaemia AND evidence of increased oxygen demand (e.g. tachyarrhythmia, hypertrophy) or reduced supply (e.g. hypotension, hypoxia or anaemia) in context of alternative clinical diagnosis
**Myocardial injury**	Myocardial necrosis (any cTnI concentration above the upper reference limit) without symptoms OR signs of myocardial ischaemia in context of alternative clinical diagnosis

The process of adjudication was conducted by two cardiologists independently. Both had access to the electronic patient record. The adjudicated diagnosis was reached by evaluating the attending clinicians documentation of the presenting complaint, past medical history, cardiovascular risk factors and clinical examination findings including routine observations (pulse, blood pressure, pulse oximetry, temperature and conscious level). All investigation results undertaken by the attending clinician were available for review, including biochemistry and haematology results, the 12 lead electrocardiogram, echocardiogram, chest X-ray and invasive coronary angiography findings when performed. Both adjudicating cardiologists had access to the final discharge letter documenting the attending clinicians' final diagnosis.

Appendix 2. R code for computation of key diagnostic metrics

```
library(binom)

#df = a data frame with pathway outcome (0 = rule out and 1 = rule in) and composite [primary outcome] (0 = no, 1 = yes).

#generate a 2x2 table
df.2x2 <- confusionMatrix(df$pathway.outcome, df$composite, positive="1")

#calculate diagnostic metrics using library(binom) and non-informative priors

#Negative predictive value (TN / TN + FN)
npv <- binom.bayes(x=df.2x2$table[1,1], n=df.2x2$table[1,1] + df.2x2$table[1,2], conf.level=0.95, type=c("central"),
prior.shape1=0.5, prior.shape2=0.5, tol=1e-9)

#Positive predictive value (TP / TP + FP)
ppv <- binom.bayes(x=df.2x2$table[2,2], n=df.2x2$table[2,1] + df.2x2$table[2,2], conf.level=0.95, type=c("central"),
prior.shape1=0.5, prior.shape2=0.5, tol=1e-9)

#Sensitivity (TP / TP + FN)
sens <- binom.bayes (x=df.2x2$table[2,2], n = df.2x2$table[1,2] + df.2x2$table[2,2], prior.shape1 = 0.5, prior.shape2 = 0.5)

#Specificity (TN / TN + FP)
spec <- binom.bayes (x=df.2x2$table[1,1], n = df.2x2$table[1,1] + df.2x2$table[2,1], prior.shape1 = 0.5, prior.shape2 = 0.5)
```
